# Traditionally used wild edible plants of district Udhampur, J&K, India

**DOI:** 10.1186/s13002-018-0272-1

**Published:** 2018-11-29

**Authors:** Harpreet Bhatia, Yash Pal Sharma, R. K. Manhas, Kewal Kumar

**Affiliations:** 1 Department of Botany, University of Jammu, Jammu, J&K 180001 India; 2 Department of Botany, Govt. Degree College, Kathua, J&K 184104 India; 3 Department of Botany, Govt. Degree College for Women, Udhampur, J&K 182101 India

**Keywords:** Cultural importance index, Traditionally used, Wild edible plants, Udhampur

## Abstract

**Background:**

Wild edible plants (WEPs) refer to edible species that are not cultivated or domesticated. WEPs have an important role to play in poverty eradication, security of food availability, diversification of agriculture, generation of income resources, and alleviating malnutrition. In the present study, an inventory of traditionally used WEPs from Udhampur district of J&K, India, has been prepared.

**Methods:**

A systematic and extensive ethnobotanical survey was carried out in different villages of the district for the collection of information on WEPs. The data collected through questionnaire and interviews was then analyzed for cultural importance index (CI) and factor informant consensus (*F*_ic_) to know the cultural significance of WEPs and consensus for the knowledge of WEPs among the informants.

**Results:**

A total of 90 plant species belonging to 45 families and 78 genera were edible and serve as wild phytofoods in the present study. Species richness of wild edible species was the maximum for vegetables (46 species) followed by fruits (37 species) and medicinal plants (36 species). Culturally (on the basis of CI), the most important vegetable and fruit species were *Diplazium esculentum*, *Fumaria indica*, *Taraxacum campylodes*, *Urtica dioica, Phyllanthus emblica*, *Punica granatum*, *Cordia dichotoma*, *Syzygium cumini*, *Ficus palmata*, etc. The highest use-report (626) was recorded for vegetables whereas the maximum mean use-report (14.8) was recorded for fruits. On an average, 20.7 wild edible species were used per informant. Informant consensus index (*F*_ic_) varied between 0.83 and 0.94 for raw vegetables and preserved vegetables, respectively.

**Conclusion:**

One of the most important issues of this era is hunger for which one of the possible solutions is the usage of WEPs. The local populace of Udhampur has good knowledge of WEPs, and this legacy of traditional culture must be conserved.

## Introduction

Wild edible plants (WEPs) refer to plant species that are not cultivated or domesticated but are accessible from various natural habitations and used as food [1]. WEPs are generally gathered from diverse habitats, viz, forests, cultivable fields, and even anthropogenically disturbed zones like roadsides and wastelands by different traditions throughout the world. A huge number of ethnic communities and local populace residing in the developing countries draw a significant part of their subsistence and livelihood from wild plants [2]. Historically, humans may have utilized more than 7000 WEPs so far [3], but many such food resources and valuable plants are still to be explored [4].

Despite the fact that most of the societies primarily rely upon agricultural crops, the tradition of utilization of WEPs has not completely vanished. According to Food and Agricultural Organization (FAO) report, at least one billion people are thought to use wild food in their diet [5]. WEPs have important role to play in poverty eradication, security of food availability, diversification of agriculture, generation of income resources [6–8], and alleviation of malnutrition [5]. The high nutrient and vitamin value of many WEPs [9–12] reduces the susceptibility of local societies to food insecurity thereby furnishing a safeguard in times of food scarcity, famine, or conflict [13–17].

WEPs have, by tradition, occupied an important position in the cultural, religious, and health sector of rural and ethnic lives of Indians. In India, the presence of varied climatic zones and ecological diversity creates a basis for rich phytodiversity and this fact is strongly supported by various studies carried out on WEPs by various researchers throughout India [18–29]. Arora and Anjula [30] have given a detailed account of WEP species occurring in India while Rathore [31] reported 600 WEP species from India. From J&K state, there are only a few studies [32–36] regarding usage of WEPs.

District Udhampur, located in Jammu division of J&K state, is a hilly terrain and many villages of the region are cut off from the frequent visits of the town. Since antiquity, the rural populace of the district has been dependent on wild plants as food because of their free availability, effectiveness against a background of undeveloped infrastructure, cultural and religious preferences, and insufficient provision of primary services. The main occupation of the local populace is agriculture. But, due to possession of small land holdings and insufficient earning, the male folk work either as laborers or are engaged in small home run shops like blacksmith and cobbler whereas the womenfolk and children are engaged in livestock rearing. On their to-and-fro journey to forests, they also collect WEPs for self-consumption and for sale in local markets as a source of income generation. The usage of WEPs has generated among them a strong base of traditional knowledge regarding phytofoods which in turn is based on their needs, instinct, observation, trial, and error coupled with experiences and has been providing them food security since antiquity. This knowledge base has developed through age-old experience and has descended orally from one generation to another as a domestic practice. But, in the present scenario, this tradition and associated knowledge is dwindling owing to developmental activities, migration from rural to urban areas for occupation and education, changing cultural traditions, attraction towards western ways of life, temptation of fast foods, declining natural resources [37–39], changing environmental conditions, deforestation, etc. [40, 41]. Balick and Cox [42] have also stated that modernization of traditions often results in the alteration of native knowledge systems as the whole community moves away from their conventional ways and adopts untraditional foreign principles. So, it is the prime need of our generation to collect and document this valuable traditional knowledge for the betterment of humanity. The present study was therefore undertaken to (i) inventorize this rich legacy of traditional knowledge available with the villagers of Udhampur district, (ii) find the cultural significance of WEPs, and (iii) evaluate consensus among the locals for the traditional knowledge of wild edible plants.

## Material and methods

### Study area

**Fig. 1:**
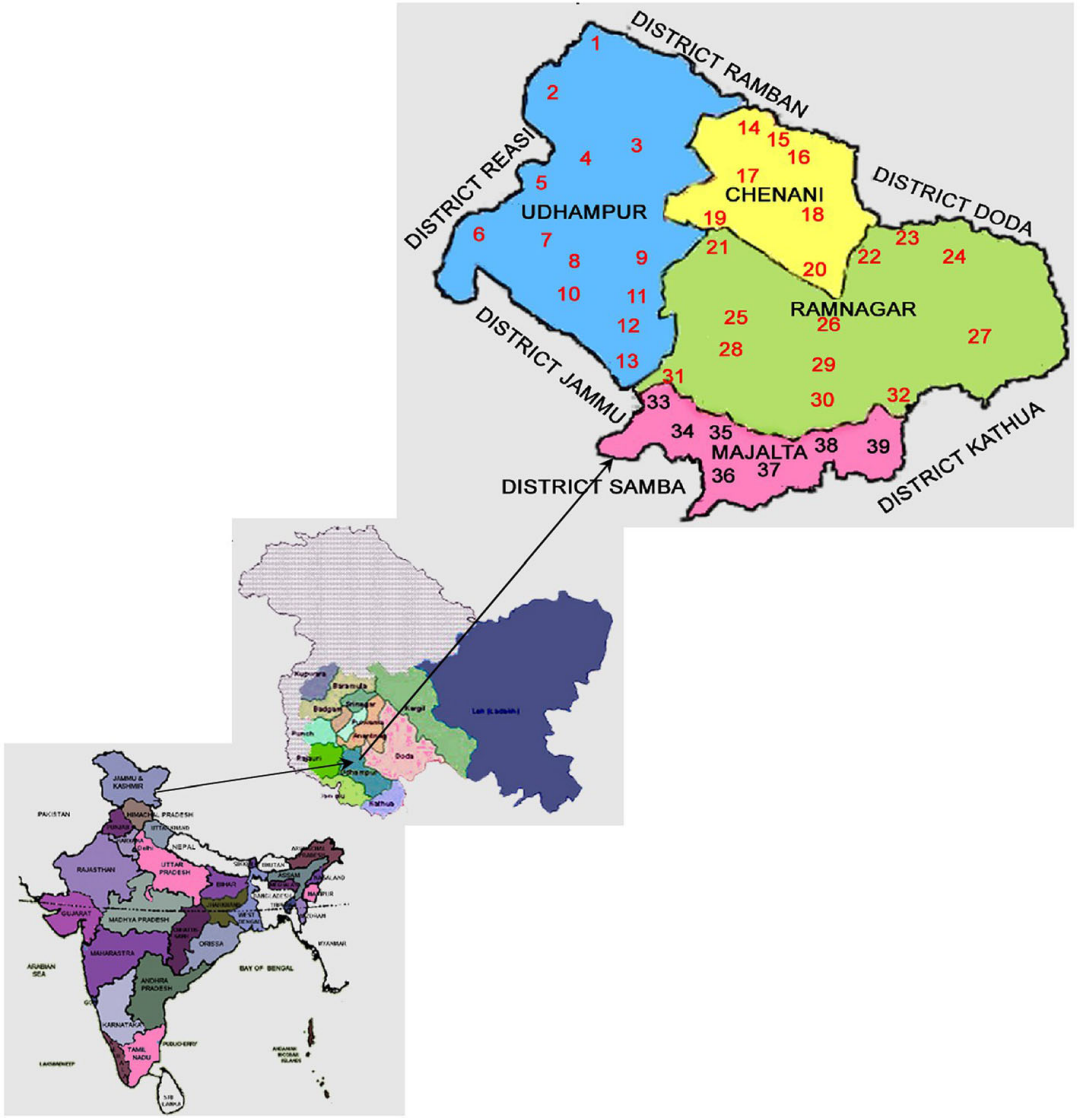
Location map of the study along with the villages (numbered 1–39) in which study was conducted

District Udhampur, located in Jammu division of J&K state, lying between 32° 34′ and 39° 30´ North latitude and 74° 16′ and 75° 38′ East longitude, has a total area of 2380 km^2^ (Fig. 1). The district is situated in the southeastern part of J&K with an altitude ranging from 600 to 2900 m above mean sea level. The topography of the district is hilly, interwoven with the Shivalik range of the Himalayas, and has largely a difficult and rugged terrain divided into three geographic zones: (a) temperate/intermediate zone, (b) sub-tropical/intermediate zone, and (c) intermediate zone. The district has an average rainfall slightly over 1551 mm [40]. Most of the rainfall takes place during July, August, and September months. Snowfall usually occurs during December to February months and nearly 25% of the study area remains snowbound during winter.

### Methodology

#### Survey and data collection

A systematic and extensive ethno-botanical survey was carried out in different villages of the district during June 2014 to June 2017 for the collection of information on wild edible plant species being used by locals in the study area. Before initiating the present study, a detailed survey was conducted during which various villages were visited and information was gathered about the people having the knowledge of wild edible plants or those involved in collection and sale of these plants. During the survey, informants were selected randomly and information was gathered by conducting interviews and group discussions with the informants in their local language on the indigenous uses of wild flora as food. A total of 88 informants (52 females and 36 males) between the age group 12–80 years were interviewed with a questionnaire. The information collected included common wild edible plant species, local name of plant species, habit, flowering time, plant part used, and recipe prepared individually or in combination with other plants.

The specimens of plant species were collected from the study site and then identified from the herbaria of the Department of Botany, University of Jammu, Jammu, and Indian Institute of Integrative Medicine, Jammu, and also with the help of various regional floras [43, 44]. The final list of the plants was prepared following the International Plant Names Index (http://www.ipni.org) and Tropicos (2017) for the botanical nomenclature of species. The plant specimens were submitted to the herbarium of the Department of Botany, University of Jammu, Jammu, J&K, India.

#### Data analysis

The wild edible plants were classified into nine categories based upon the local usage and recipes (Table 1). The vegetable use category was further subcategorized as cooked (Veg_C_), raw (Veg_R_), and preserved (Veg_P_). The vegetables which are consumed after cooking, grinding or boiling, and mixing with yogurt were categorized as cooked whereas preparations preserved in mustard oil and consumed as food during shortage period were classified as preserved and those consumed directly after washing or as salad were designated as raw. Fruit usage was subcategorized as raw (Fr_R_) and processed (Fr_P_). Due to less number of fruit species, both cooked and preserved subcategories were merged as processed. Other categories included spices (Sp), beverages (Bv), and medicinal plants (Med).

**Table 1:** Edible usages and culture index of wild edible species of Udhampur district

Botanical name	Family	Voucher no. (JUH)	Local name	Life form	Plant part used	Edible usage	CI
*Acacia catechu* (L. f.) Willd.	Leguminosae	14552	*Katha*	Tree	Heartwood, branches	Tea; medicinal.	0.23
*Aegle marmelos* (L.) Correa	Rutaceae	14594	*Bel patri*	Tree	Fruits	Fruit pulp is eaten fresh. Fruits are eaten raw and infusion is sieved for making *sharbat* (cooling drink); fruit is used as medicine.	0.34
*Agave americana* L.	Asparagaceae	14605	*Kyora*	Shrub	Flowers	Vegetable, pickled.	0.10
*Amaranthus viridis* L.	Amaranthaceae	14452	*Chaleree*	Herb	Leaves	Vegetable; medicinal.	0.28
*Artemisia scoparia* Waldst. & Kitam.	Compositae	14606	*Chaau*	Herb	Seeds, flowers, leaves	Condiment, spice; medicinal.	0.34
*Artocarpus lacucha* Buch.-Ham.	Moraceae	14607	*Taeoo*	Tree	Fruits	Pickled.	0.30
*Asparagus adscendens* Roxb.	Asparagaceae	14470	*Sanspour*	Herb	Tubers	Vegetable, pickled, used as salad; medicinal.	0.31
*Bauhinia vahlii* Wight & Arn.	Leguminosae	14493	*Malungarh*	Climber	Seeds	Seeds are consumed after roasting/frying; flower buds are added to yogurt to make *raita* or *aasara*, pickled.	0.13
*Bauhinia variegata* L.	Leguminosae	14494	*Kartair*	Tree	Flowers	Vegetable is prepared in yogurt; flower buds are added to yogurt to make *raita* or *aasara*, pickled.	0.17
*Berberis lycium* Royle	Berberidaceae	14484	*Kambel*	Shrub	Fruits	Fruits are eaten raw; root is medicinal.	0.43
*Bombax ceiba* L.	Malvaceae	14608	*Simbal*	Tree	Flower receptacle	Vegetable.	0.15
*Buglossoides arvensis* (L.) I. M. Johnst.	Boraginaceae	14609	*Bibru*	Herb	Fruits	Fruits are eaten raw.	0.10
*Capsella bursa–pastoris* (L.) Medik.	Brassicaceae	14692	*Chiri halian*	Herb	Leaves	Vegetable.	0.08
*Cardamine impatiens* L.	Brassicaceae	14870	*Daraati*	Herb	Leaves	Vegetable.	0.08
*Carissa spinarum* L.	Apocynaceae	14463	*Garna*	Shrub	Fruits	Fruits are eaten raw.	0.22
*Carum carvi* L.	Apiaceae	14940	*Shia jeera*	Herb	Fruits, seeds	Spice, condiment.	0.07
*Celastrus paniculatus* Willd.	Celastraceae	14610	*Sankhir*	Shrub	Fruits	Fruits are eaten raw.	0.09
*Chenopodium album* L.	Amaranthaceae	14504	*Bathu*	Herb	Leaves	Vegetable.	0.16
*Cissampelos glaberrima* A. St.-Hill.	Menispermaceae	14550	*Battal bel*	Climber	Leaves	*Pakora* (prepared by deep frying leaves coated with wheat flour), *chutney* (prepared by grinding leaves); medicinal.	0.40
*Citrus aurantiifolia* (Christm.) Swingle	Rutaceae	14611	*Jambiri*	Tree	Fruits	Juice is added to chutneys, eaten raw.	0.23
*Citrus medica* L.	Rutaceae	14596	*Gargal*	Tree	Fruits	Pickled, juice is added to chutneys; medicinal.	0.40
*Colocasia esculenta* (L.) Schott	Araceae	14612	*Kachaloo*	Herb	Tubers	Boiled and eaten with spices and juice of *Citrus medica*; cooked as vegetable.	0.20
*Commelina benghalensis* L.	Commelinaceae	14505	*Chura*	Herb	Leaves	Vegetable.	0.05
*Cordia dichotoma* G. Forst.	Boraginaceae	14613	*Lasoora*	Tree	Fruits	Vegetable and pickled.	0.51
*Cucumis melo var. utilissimus* (Roxb.) Duthie & Fuller	Cucurbitaceae	14614	*Tar*	Climber	Fruits	As salad.	0.17
*Cydonia oblonga* Mill.	Rosaceae	14615	*Bai dana*	Tree	Fruits	Fruits are eaten raw, *murabba* (prepared by shade drying boiled fruits and preserving them in sugar).	0.26
*Dioscorea belophylla* (Prain) Voigt ex Haines	Dioscoreaceae	14616	*Tarar bail*	Herb	Tubers	Vegetable, pickle; medicinal.	0.24
*Diospyros lotus* L.	Ebenaceae	14617	*Amlook*	Tree	Fruits	Dried fruits are eaten; medicinal.	0.16
*Diplazium esculentum* (Retz.) Sw.	Athyriaceae	14618	*Kasroor*	Herb	Fronds	Vegetable, pickled.	0.32
*Duchesnea indica* (Jacks.) Focke	Rosaceae	14619	*Jangali shatawari*	Herb	Fruits	Fruits are eaten raw; medicinal.	0.09
*Elaeagnus parvifolia* wall. ex Royle	Elaeagnaceae	14620	*Kaain*	Shrub	Fruits	Fruits are eaten raw.	0.02
*Euphorbia royleana* Boiss.	Euphorbiaceae	14515	*Thor*	Shrub	Young shoots	Young shoots are boiled, cut into small pieces, and added to yogurt to make *raita* or *aasra*; medicinal.	0.07
*Fagopyrum acutatum* (Lehm.) Mansf. ex K. Hammer	Polygonaceae	14621	*Van daraiyoon*	Herb	Leaves, seeds	Vegetable; chapatti of flour are eaten during severe cold.	0.06
*Ficus auriculata* Lour.	Moraceae	14622	*Trimbal*	Tree	Fruits	Fruits are eaten raw.	0.06
*Ficus palmata* Forssk.	Moraceae	14557	*Phagwara*	Tree	Young leaves, fruits	Young leaves are cooked with diluted yogurt to make curry, leaves are also cooked with meat; fruits are eaten raw; medicinal.	0.43
*Ficus racemosa* L.	Moraceae	14558	*Rumbal*	Tree	Fruits	Fruits are eaten raw.	0.15
*Flacourtia indica* (Burm. f.) Merr.	Salicaceae	14623	*Kakooaa*	Tree	Fruits, inner bark	Fruits are eaten raw; tea.	0.19
*Flemingia prostrata* Roxb.	Leguminosae	14624	*Titri*	Shrub	Fruits, seeds	*Chutney* (prepared by grinding fruits and seeds).	0.06
*Fumaria indica* (Hausskn.) Pusley	Papaveraceae	14528	*Pitpapra, indu*	Herb	Leaves	Vegetable; medicinal.	0.51
*Impatiens bicolor* Royle	Balsaminaceae	14625	*Alwa*	Herb	Seeds	Fruits are eaten raw.	0.03
*Indigofera cassioides* DC.	Leguminosae	14626	*Kathi*	Shrub	Flowers	Vegetable.	0.05
*Juglans regia* L.	Juglandaceae	14531	*Akhrot*	Tree	Fruits	Fruits are eaten raw, *chutney* is prepared; medicinal.	0.30
*Lamium amplexicaule* L.	Lamiaceae	14627	*Indu saag*	Herb	Leaves, young shoots	Vegetable.	0.03
*Lathyrus aphaca* L.	Leguminosae	14628	*Khand khiru, mithu saag*	Herb	Leaves, young shoots, seeds	Vegetable, young seeds are eaten; fruits are eaten raw.	0.15
*Malva parviflora* L.	Malvaceae	14546	*Sonchal*	Herb	Leaves	Vegetable.	0.20
*Medicago polymorpha* L.	Leguminosae	14740	*Sriri*	Herb	Leaves	Vegetable; medicinal.	0.34
*Mentha longifolia* (L.) L.	Lamiaceae	14535	*Mainani*	Herb	Leaves	*Chutney*; tea; medicinal.	0.70
*Moringa oleifera* Lam.	Moringaceae	14629	*Sohanjana*	Tree	Pods, roots	Vegetable, pickle; medicinal.	0.35
*Morus alba* L.	Moraceae	14560	*Shtoot*	Tree	Fruits	Fruits are eaten raw; medicinal.	0.28
*Morus nigra* L.	Moraceae	14872	*Kala toot*	Tree	Fruits	Fruits are eaten raw; medicinal.	0.17
*Murraya koenigii* (L.) Spreng.	Rutaceae	14630	*Kari patta, drankari*	Shrub	Leaves	Spice; medicinal.	0.14
*Nelumbo nucifera* Gaertn.	Nelumbonaceae	14631	*Nadroo*	Herb	Roots	Vegetable is prepared either with tomato puree or yogurt (dish is locally called *nadroo ki yakhni*)	0.06
*Nepeta laevigata* (D. Don) Hand.-Mazz.	Lamiaceae	14632	*Kaali khari*	Herb	Fruits	Fruits are eaten raw.	0.03
*Ocimum basilicum* L.	Lamiaceae	14536	*Babbari, Naazposh*	Herb	Seeds	*Sharbat* (cooling drink prepared as infusion or decoction); medicinal.	0.03
*Oxalis corniculata* L.	Oxalidaceae	14569	*Ammi*	Herb	Leaves	Chutney; medicinal.	0.08
*Persicaria amplexicaulis* (D. Don) Ronse Decr.	Phyllanthaceae	14580	*Aambla*	Tree	Fruits	Tea.	0.06
*Phoenix sylvestris* (L.) Roxb.	Arecaceae	14633	*Khajoor*	Tree	Fruits	Fruits are eaten raw.	0.10
*Phyllanthus emblica* L.	Plantaginaceae	14517	*Challa*	Herb	Leaves	Fruits are eaten raw, pickle, *murabba*; medicinal.	0.86
*Plantago lanceolata* L.	Polygonaceae	14725	*Masloon*	Herb	Roots	Vegetable.	0.03
*Prunus armeniaca* L.	Rosaceae	14587	*Saari, haari*	Tree	Fruits	Fruits are eaten raw.	0.30
*Prunus persica* (L.) Batsch	Rosaceae	14588	*Aaru, aarn*	Tree	Fruits	Fruits are eaten raw.	0.33
*Pueraria tuberosa* (Willd.) DC.	Leguminosae	14634	*Bidda*	Climber	Tubers	Fruits are eaten raw and cooked as vegetable.	0.10
*Punica granatum* L.	Lythraceae	14542	*Darooni*	Tree	Fruits, seeds	Fruits are eaten raw, *anaardana* (dried seeds) are ground to make *chutney*; medicinal.	0.68
*Pyrus pashia* Buch.-Ham. ex D.Don	Rosaceae	14589	*Batangi, kainth*	Tree	Fruits	Fruits are eaten raw.	0.02
*Ranunculus arvensis* L.	Ranunculaceae	14858	*Tilphari*	Herb	Whole plant	Whole plant in vegetative stage is washed, sun dried, and oil and *tatri* are added and consumed as pickle.	0.03
*Rosa indica* L.	Rosaceae	14590	*Gulaab*	Shrub	Petals	*Gulukand* (prepared by preserving petals in sugar); medicinal.	0.15
*Rubus ellipticus* Sm.	Rosaceae	14592	*Aakhe*	Shrub	Fruits	Fruits are eaten raw.	0.18
*Rumex dentatus* L.	Polygonaceae	14581	*Tandalak*	Herb	Leaves	Vegetable.	0.07
*Rumex hastatus* D. Don	Polygonaceae	14582	*Ammi*	Herb	Leaves	Vegetable, *chutney*.	0.22
*Rumex nepalensis* Spreng.	Polygonaceae	14635	*Urval*	Herb	Leaves	Vegetable, curry made in yogurt.	0.27
*Scandix pecten-veneris* L.	Apiaceae	14636	*Bhuss, indu saag*	Herb	Young shoots	Vegetable.	0.02
*Silene conoidea* L.	Caryophyllaceae	14502	*Takla*	Herb	Leaves, fruits	Vegetable; fruits are eaten raw.	0.13
*Stellaria media* (L.) Vill.	Caryophyllaceae	14850	*Koukoon, laadroon*	Herb	Leaves, shoots	Vegetable.	0.02
*Syzygium cumini* (L.) Skeels	Myrtaceae	14564	*Tallay*	Tree	Fruits	Fruits are eaten raw; medicinal.	0.57
*Taraxacum campylodes* G. E. Haglund	Compositae	14480	*Phul dudhli*	Herb	Leaves	Vegetable; medicinal.	0.50
*Telosma cordata* (Burm. F.) Merr.	Apocynaceae	14637	*Guaal manda*	Climber	Flowers	Vegetable, eaten raw.	0.14
*Terminalia arjuna* (Roxb. ex DC.) Wight & Arn.	Combretaceae	14638	*Arjan*	Tree	Fruits	Fruits are eaten raw; medicinal.	0.15
*Terminalia bellirica* (Gaertn.) Roxb.	Combretaceae	14639	*Bahera*	Tree	Fruits	Fruits are eaten raw after removing outer covering; medicinal.	0.41
*Terminalia chebula* Retz.	Combretaceae	14640	*Reer*	Tree	Fruits	Vegetable, curry; medicinal.	0.22
*Trifolium pratense* L.	Leguminosae	14774	*Shataala*	Herb	Leaves, shoots	Vegetable; medicinal.	0.11
*Trifolium repens* L.	Leguminosae	14798	*Chaptal*	Herb	Leaves	Vegetable; medicinal.	0.08
*Tulipa clusiana* DC.	Liliaceae	14697	*Maghey, kayaloon, kukarboona*	Herb	Tubers	Tubers are eaten raw.	0.02
*Urtica dioica* L.	Urticaceae	14772	*Saddar*	Herb	Young leaves and shoots	Vegetable; beverage; medicinal.	0.49
*Veronica persica* Poir.	Plantaginaceae	14754	*Titi*	Herb	Young leaves and shoots	Vegetable is prepared in yogurt.	0.05
*Viburnum grandiflorum* Wall. ex DC.	Adoxaceae	14641	*Taildi*	Shrub	Fruits	Fruits are eaten raw.	0.02
*Vicia hirsuta* (L.) Gray	Leguminosae	14642	*Kanghi*	Herb	Pods	Pods are eaten raw.	0.01
*Vicia sativa* L.	Leguminosae	14934	*Jawaal*	Herb	Leaves, young shoots, seeds	Vegetable is prepared from leaves; dried seeds are cooked as pulse; fresh seeds are eaten raw.	0.09
*Viola odorata* L.	Violaceae	15752	*Banafsha*	Herb	Flowers	Tea; medicinal.	0.39
*Zanthoxylum armatum* DC.	Rutaceae	14597	*Timbru*	Shrub	Seeds, fruits	*Chutney* is prepared from seeds; fruits are sun dried, fried in oil, and used as pickle; medicinal.	0.42
*Zizyphus jujuba* Mill.	Rhamnaceae	14767	*Ber*	Tree	Fruits	Fruits are eaten raw; medicinal.	0.27

The field information gathered through questionnaire and interviews was analyzed quantitatively using two ethnobotanical indices described as follows.

##### Factor informant consensus (*F*_ic_)

To test the homogeneity of knowledge about the medicinal plants, the factor informant consensus (*F*_ic_) was used [45]. The *F*_ic_ was calculated as:

$$ {F}_{\mathrm{ic}}=\frac{n_{\mathrm{ur}}-{n}_{\mathrm{t}}}{n_{\mathrm{ur}}-1} $$

where *n*_ur_ refers to the number of use-reports for a particular use category and *n*_t_ refers to the number of taxa used for a particular use category by all the informants. *F*_ic_ values are low (near 0) if plants are chosen randomly or if there is no exchange of information about their use among informants, and approach one (1) when there is a well-defined selection criterion in the community and/or if information is exchanged between informants [46, 47].

##### Cultural importance index (CI)

The cultural importance index (CI) is defined by the following formula [48]:

$$ {\mathrm{CI}}_{\mathrm{s}}=\left[{\Sigma}_{u={u}_1}^{u_{\mathrm{NC}}}{\Sigma}_{i={i}_1}^{u_N}U{R}_{ui/N}\right] $$

CI index can also be seen as the sum of the proportion of informants that mention each species use. This additive index takes into account not only the spread of the use (number of informants) for each species but also its versatility, i.e., the diversity of its uses. The theoretical maximum value of the index is the total number of different use-categories (NC), reached in the unlikely case that all the informants would mention the use of the species in all the use-categories considered in a survey. In the case of species with only one use, this index would be equal to RFC [48].

## Results and discussion

### Informants

Wild edible plants remain a significant source of food and income for many countryside populations of the world. In the present study, the main occupation of the local populace is agriculture. But due to small land holdings, the male folk work either as laborers or are engaged in small home run shops, blacksmiths, cobblers, etc. Knowledge about wild vegetables and their recipes was mainly confined to women folk (86.4%). This unequal distribution of knowledge owes to the fact that collection of WEPs was mainly done by them during agricultural activities and on their to-and-fro journey to fields and forests for livestock-rearing activities. Gender is a crucial variable that influences the traditional knowledge of an area because it is highly correlated with numerous sociocultural factors like livelihood, education, accessibility to resources, status, and networking in the society [49]. Women of every society tend to have an edge over these sociocultural factors and hence their knowledge is much more than others [50]. As evident from the present study, women are usually unemployed in these far-flung areas and fully dedicate themselves to household and other cattle-related activities. They combine this day-to-day information with culturally attained knowledge to enhance their subsistence [51].

### Diversity of wild edible plants

A total of 90 plant species (89 angiosperms and 1 pteridophyte, viz, *Diplazium esculentum*) belonging to 45 families and 78 genera serve as wild phytofoods in Udhampur (Table 1). Out of the 89 species of flowering plants, 95.5% (85 species) belong to dicots and 4.5% (4 species, viz, *Commelina benghalensis*, *Tulipa clusiana*, *Colocasia esculenta*, and *Phoenix sylvestris*) belong to monocots. Singh et al. [52] have reported 111 wild edible plants from Kashmir Himalayas whereas Thakur et al. [8] have recorded 50 phytofoods from tribal areas of Western Himalaya. Some other studies from different parts of the world have reported 49 to 173 wild edible plants [53–55]. The high usage of wild plants as vegetables and fruits, in the present study, is an indicator of rich diversity of plants, easy availability, deep knowledge of wild edible plants, day-to-day requirements, well-maintained forests, far-off residential places from the local markets, and/or poor economic status of the local populace.

**Fig. 2:**
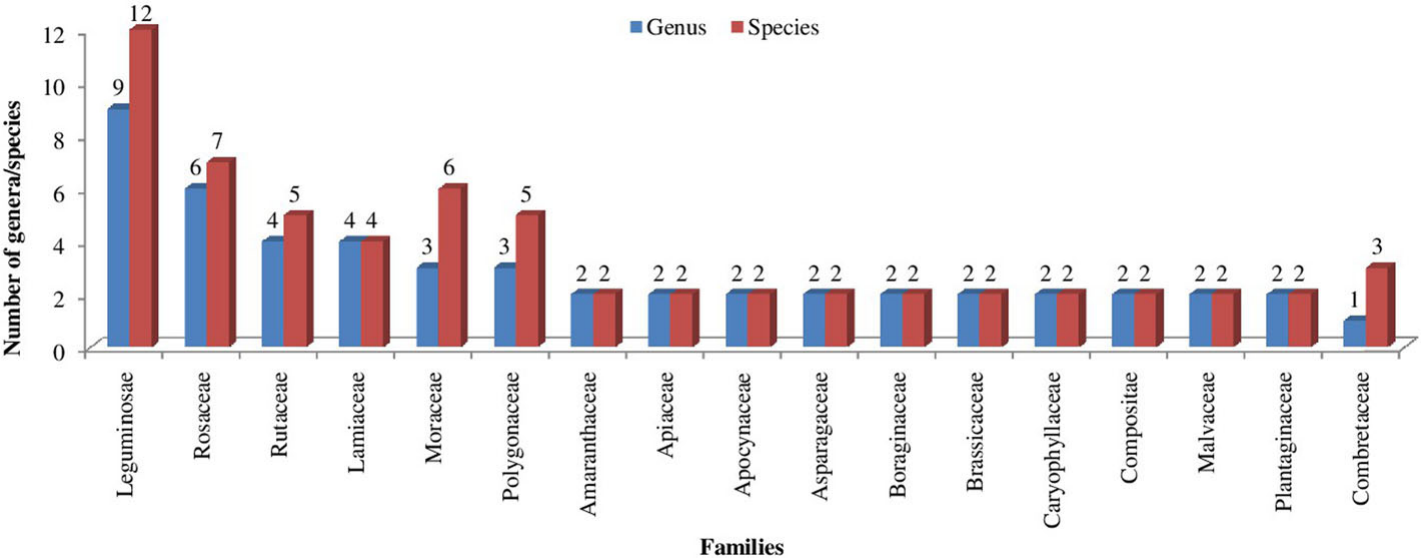
Most represented wild plant families and number of genera/species in Udhampur

In terms of number of species used, Leguminosae was the most dominant family (12 species) followed by Rosaceae (7 species), Moraceae (6 species), Rutaceae and Polygonaceae (5 species each), Lamiaceae (4 species), and Combretaceae (3 species) (Fig. 2). Twenty-eight families were represented by a single species each. In contrast to Rosaceae, being the most dominant family used in other studies worldwide [8, 52, 54, 55], Leguminosae was the most important family in the present study. This may be ascribed to the dominance of Leguminosae in the local flora or higher relevance of vegetables in the day-to-day life of the local populace of Udhampur. Pardo-de-Santayana et al. [53] have reported Lamiaceae as the most important plant family in Montesinho, Portugal, owing to the higher significance of condiments in the area.

Approximately, 20.7 wild edible species were used per informant in the present study. Highest value of usage was recorded for vegetable (mean 7.2), followed by fruits (mean 6.2), and food medicine plants (mean 5.0). Thakur et al. [8] have recorded 23.7 species per respondent in the tribal areas of Himachal Pradesh, India; Geng et al. [55] have recorded an average of 20.6 taxa per informant by the Naxi in northwest Yunnan, China; Kang et al. [56] have stated an average of 20.8 wild edible species per informant from Gansu province of China, and Dolina et al. [57] have reported a mean of 13.2 and 14.6 species per informant in Poljica and Krk areas of Croatia, respectively. In all these studies, the mean number of vegetable species per informant ranged between 7.1 and 13.2 and the mean number of fruit species per respondent between 6.3 and 6.9. The values reported in the present study are well within these ranges for total wild edible species, vegetable and fruit species.

### Informant consensus index (*F*_ic_)

Informant consensus index (*F*_ic_) varied between 0.83 for raw vegetables and 0.94 for preserved vegetables (Table 2). Wild fruits eaten raw and *chutney* preparations also recorded high values (0.93 each) for *F*_ic_. Similar results of high *F*_ic_ values have also been reported by Rao et al. [38], Bhatia et al. [40], Bhatia et al. [41], Singh et al. [52], and Kumar et al. [58] from various parts of Jammu and Kashmir owing to high level of sharing of indigenous knowledge among the informants. These findings point towards the fact that despite huge variation in communities, climatic conditions, and forests, informants have good knowledge of WEPs which is being shared to a great extent among the inhabitants and also wild phytofoods are presently in use among the local populace.

**Table 2:** Species richness and cultural importance of various use-categories and subcategories of wild edible plants

Use-category/subcategories	Number of species	Use-report (UR)	Mean UR	CI	*F*_ic_
Vegetables	46	626	13.6	7.1	0.93
Vegetable (cooked)	43	457	10.6	5.2	0.91
Vegetable (raw)	4	19	4.8	0.2	0.83
Vegetable (preserved)	10	150	15.0	1.7	0.94
Fruits	37	547	14.8	6.2	0.93
Fruit (processed)	9	73	8.1	0.8	0.89
Fruit (raw)	35	474	13.5	5.4	0.93
Chutney	10	138	13.8	1.6	0.93
Spices	3	21	7.0	0.2	0.90
Beverage	6	46	7.7	0.5	0.89
Medicinal WEPs	36	436	12.1	5.0	0.92

### Usages of wild edible plants and cultural importance

**Fig. 3:**
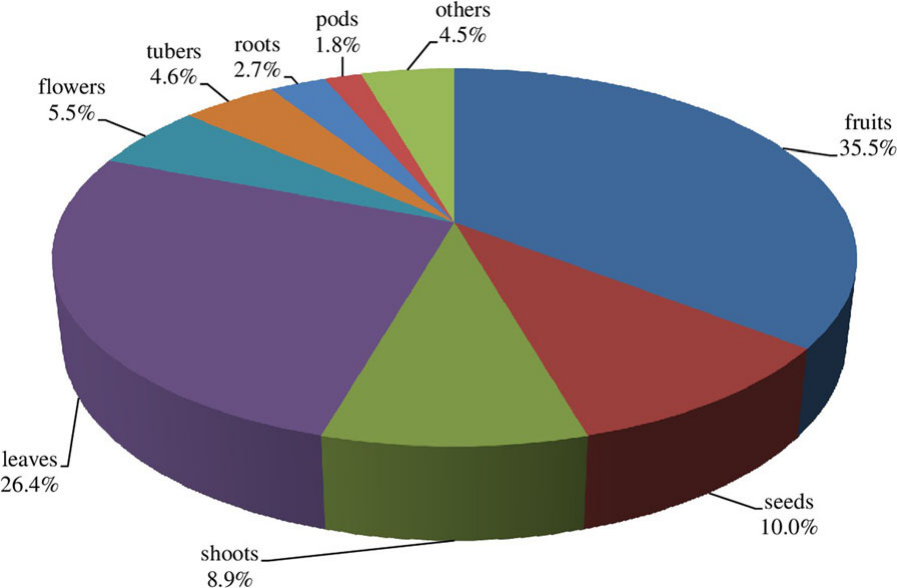
Plant part of WEPs used. Other plant parts (4.5%) include heartwood, flower receptacle, fronds, inner bark, petals, and whole plant

**Fig. 4:**
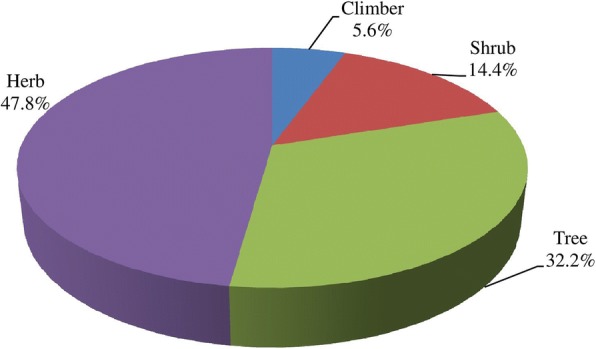
Habit of WEPs

The present study site is having rich diversity of vegetables, fruits, and medicinal plants (Table 2). Species richness of WEPs was the maximum for vegetables (46 species) followed by fruits (37 species) and medicinal plants (36 species). Vegetables were mainly consumed after cooking (93.5%) and fruits as raw (94.6%). Highest use-report (626) was recorded for vegetables whereas maximum mean use-report (14.8) was recorded for fruits. Edible value was not confined to one or more plant parts. Fruits were the edible part in majority of the cases (35.5%) followed by leaves (26.4%), seeds (10.0%), shoots (8.9%), flowers (5.5%), tubers (4.6%), roots (2.7%), and pods (1.8%) (Fig. 3). The wild edibles were mainly herbs (47.8%, 43 species) followed by trees (32.2%, 29 species), shrubs (14.4%, 13 species), and climbers (5.6%, 5 species) (Fig. 4).

The cultural index value for vegetables was 34.9% of the total CI, closely followed by fruits (30.5%) and medicinal plants (24.3%). Culturally (on the basis of CI), the most important vegetable species were *Diplazium esculentum* (0.52), *Fumaria indica* (0.51), *Taraxacum campylodes* (0.50), *Urtica dioica* (0.49), *Zanthoxylum armatum* (0.42), etc. The most common fruits consumed by the locals were *Phyllanthus emblica* (CI, 0.94), *Punica granatum* (CI, 0.68), *Cordia dichotoma* (CI, 0.60), *Syzygium cumini* (CI, 0.57), *Ficus palmata* (CI, 0.51), *Berberis lycium* (CI, 0.43), *Prunus armeniaca* (CI, 0.33), *Prunus persica* (CI, 0.31), *Pyrus pashia* (CI, 0.22), and *Rubus ellipticus* (CI, 0.18).

### Vegetables

Most of the species with high CI in the present study are also used as vegetable in most parts of the Himalayas [8, 15, 52, 59]. The immature fronds of *Diplazium esculentum* are either cooked as vegetable or preserved as pickle in the study area, same as in other parts of the Himalayas [8, 15, 52, 59–61]. According to Zeghichi et al. [62], vegetables gathered from the wild have diverse, potentially more nutrients than the commercially cultivated species. Seal [63] has reported the leaves of *Diplazium esculentum* with high moisture content to have good nutritive value (3413.2 Kcal Kg^−1^) and crude protein content (143.8 g Kg^−1^). The values of protein content are even higher than many commercial fruits and leafy vegetables like apple, litchi, cabbage, and cauliflower [63, 64]. *Taraxacum campylodes* as vegetable is a rich source of proteins, calcium, phosphorus, and dietary fiber [65]. *Urtica dioica* has a huge local value as vegetable in India and other parts of the world [8, 37, 52, 66–68]. It is a good source of vitamin A, dietary calcium, iron, crude proteins, fiber, fat, and carbohydrates [68, 69].

*Nelumbo nucifera* (CI, 0.06) grows in ponds, lakes, and marshy and swampy areas. Fresh rhizome of this plant contains 83.8% water, 0.1% fat, 1.6% reducing sugar, 0.4% sucrose, 2.7% crude protein, 9.3% starch, 0.8% fiber, l.1% ash, and 0.1% calcium [70]. Rhizomes/petioles of *Nelumbo nucifera* are eaten as vegetable prepared either in tomato puree or in yogurt; the dish prepared in yogurt is locally known as *nadroo ki yakhni* and is considered a delicacy during marriages and other festive occasions. A combination of yogurt and vegetables is also common in many Middle East countries [71].

A good number of vegetables are sun-dried or preserved as pickle (10 species). The sun-dried vegetables are meant for usage in winters, the period of scarcity, especially in the hilly zones. This is again a tradition practiced in the Himalayas; some plants are preserved in mustard oil and salt in the form of pickles, viz, young fronds of *Diplazium esculentum* [8, 52, 61], pods and roots of *Moringa oleifera* [35, 72], flowers of *Agave americana* [73], and whole plant of *Ranunculus arvensis*.

### Fruits

Fruits are mainly consumed raw. Some of the most common fruits consumed by the locals were *Phyllanthus emblica*, *Punica granatum*, *Cordia dichotoma*, *Syzygium cumini*, *Ficus palmata*, *Berberis lycium*, *Prunus armeniaca*, *Prunus persica*, *Pyrus pashia*, *Rubus ellipticus*, etc. Arya [74] has also reported *Rubus ellipticus*, *Pyrus pashia*, *Elaeagnus parvifolia*, *Carissa spinarum*, and *Ficus palmata* to be wild edible fruits used by the locals of Garhwal Himalaya, India. Thakur et al. [8] have also recorded the usage of *Prunus armeniaca*, *Pyrus pashia*, *Pyrus pashia*, and *Rubus ellipticus* as raw fruits from the tribal area of Western Himalaya. As per Johns [75], wild fruits contain more fiber and have higher concentrations of vitamins and greater diversity of secondary metabolites in comparison to cultivated species.

Fruit pulp of *Aegle marmelos* and seeds of *Ocimum basilicum* are used for preparation of traditional beverage or drink (locally known as *Sharbat*) during summers. Both *Aegle marmelos* [76] and *Ocimum basilicum* [77] are used as drinks in various parts of India and other neighboring countries. Fruits of *Phoenix sylvestris*, being calorie-rich and having numerous vital and refreshing compounds, are consumed worldwide especially by Muslims during the holy month of Ramadan to break the day-long fast [78].

**Fig. 5:**
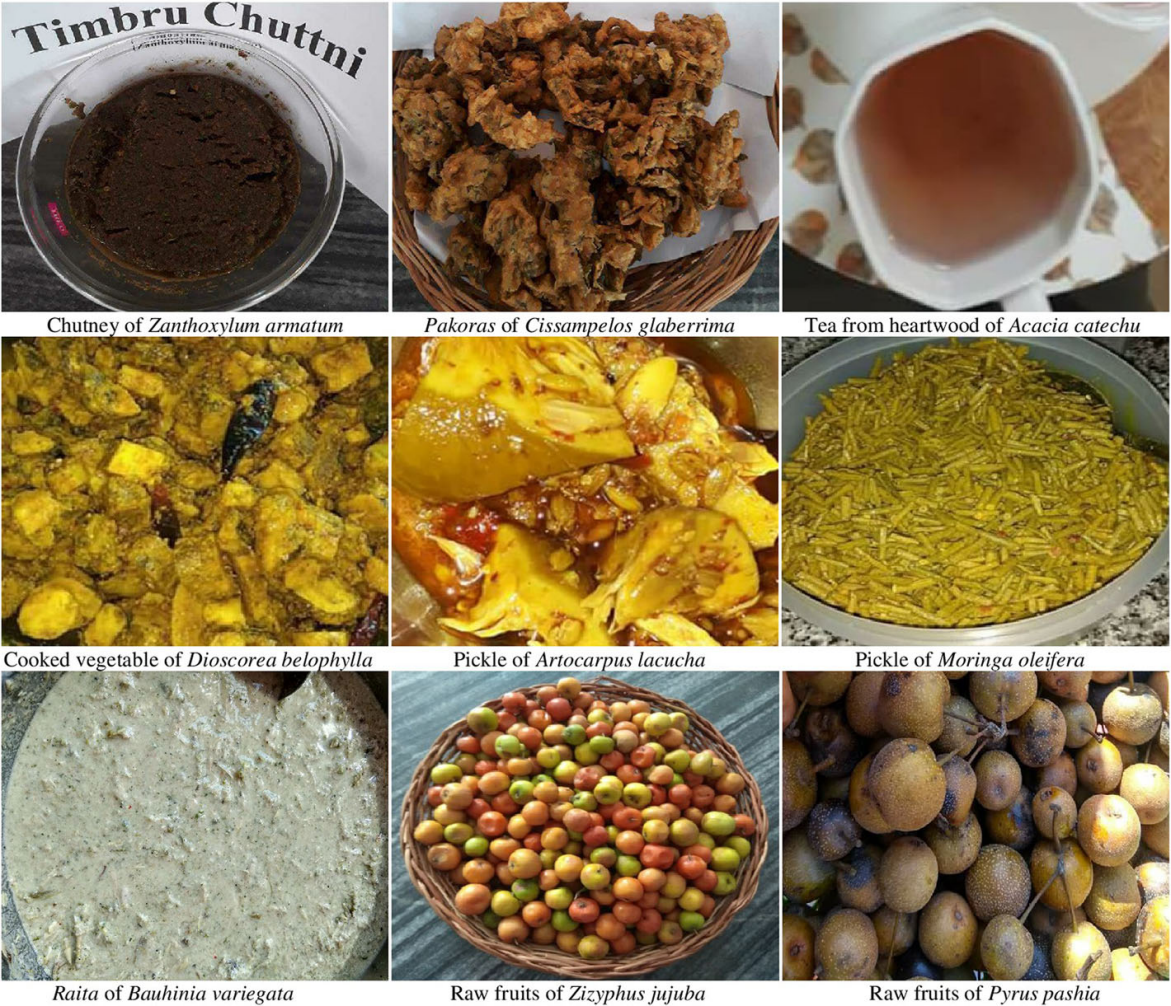
Photographs of some recipes and wild raw fruits from Udhampur

Some fruit species, viz, *Cydonia oblonga* and *Phyllanthus emblica*, are preserved for months or even years in the form of a local preparation called *murabba* (local jam) prepared by boiling whole or sliced fruits followed by shade drying and storing in airtight containers containing sugar or sugar syrup. Fruits of *Artocarpus lacucha* (Fig. 5), *Phyllanthus emblica*, and *Citrus medica* are preserved in mustard oil along with spices and salt as pickle. All these practices of preservation of fruits for the period of scarcity are part of local culture and also practiced in various other parts of India and in the Himalayas [8].

### Food medicine

It is a well-known fact that wild edibles are used as medicines worldwide [54], and in general, these plants are called as nutraceuticals [79]. A good number of the wild edibles were reported as food medicine in the present study. They stand third, after the vegetable and fruits, in all respects as number (36 species), citations (436), and citations per informant (5.0). These species that can be a wild vegetable or fruit are consumed as per the availability or on special occasions like locals cook the leaves of *Taraxacum campylodes* (CI, 0.50) as vegetable especially as pre- and post pregnancy food for ladies to overcome weakness. It is a rich source of minerals particularly potassium [80] as well as protein, boron, calcium, choline, copper, iron, manganese, magnesium, potassium, silicon, and zinc [81] and a rich source of vitamins A, B complex, C, and D [40, 58, 82]. *Urtica dioica* (CI, 0.49) is a multi-utility medicinal species [38]. The plant is diuretic and its tender leaves are picked up and cooked as local delicacy. *Cissampelos glaberrima* (CI, 0.40) is consumed only at the time of diarrhea [40]. Jam (*gulukand*) prepared from the petals of rose after adding sugar is used as medicine to cure mouth ulcers and improve digestion and use against jaundice.

### Chutney

On an average, 1.6 wild edible species per informant were mentioned to be utilized for the preparation of chutney. Leaves of *Mentha longifolia* are grinded separately in mortar and pestle with subsequent addition of salt and spices resulting in the preparation of *chutney*. It is a ready-to-eat food for instant consumption and generally referred to as the poor man’s food adjuster [83]. *Chutney* is part of daily meal especially during summer season as it is a very good appetizer, antigastric, and antispasmodic and improves digestion [38, 40].

Seeds of *Punica granatum*, *Zanthoxylum armatum* (Fig. 5), and *Flemingia prostrata* are also consumed as *chutney*. This recipe improves digestion and is a good appetizer [40]. Ramachandran and Ali [84] also reported the fruit and seeds of *Zanthoxylum armatum* to be consumed as aromatic and tonic, in fever and dyspepsia and in expelling round worm.

### Beverages

In the study area, four species, viz, *Viola odorata* (flowers), *Persicaria amplexicaulis* (roots), *Flacourtia indica* (inner bark), and *Acacia catechu* (heartwood) are boiled in hot water and serve as tea substitutes (hot drinks) after adding sweeteners. Tea prepared from flowers of *Viola odorata* is used against cough, cold, fever, and jaundice. *Viola odorata* contains alkaloids, mucilage, and vitamin C [85] having diuretic [86] and antioxidant properties [87] and used against bronchitis, cancer, cold and cough, fever, kidney troubles, liver disorders, rheumatism, sneezing, and urinary infections [88, 89].

### Spices

Owing to their aromatic properties, three species, namely *Artemisia scoparia* (seeds, flowers, and leaves), *Carum carvi* (fruits, seeds), and *Murraya koenigii* (leaves), are added to pulses and vegetables as condiment and spice in the study area. *Carum carvi* is a valuable spice found in the wild all through Europe, Russia, Siberia, and the Himalayas [90], having antibacterial, antiproligerative, antifungal, antitumor, and antihyperglycemic properties [91]. The aromatic leaves of *Murraya koenigii* are highly valued, in different parts of Asia, for their utility as condiment and spice [60, 92–94] and for medicinal properties such as antidiabetic, antidysenteric, antioxidant, anti-inflammatory, anticarcinogenic, and hepatoprotective [93, 94].

## Conclusions

Hunger, one of the most important concerns of this generation, can be supplemented, to a great extent, by the inclusion of WEPs in diet. Present study revealed that the traditional knowledge about the use of WEPs is still in practice among the ethnic communities of the study area. High diversity of vegetable, fruit, and food medicine plants in use along with greater consensus for their usage also supports this statement. The informants depend on these resources, developed in an agricultural and pastoral context, not only for food and nutrition but also for income generation. The culturally important plants of the study site are also common in use in other parts of the Himalayas which shows that broadly the dweller of the great Himalayan range have some similarities in their traditions. Persistence with the conventional foods is a powerful tool in the conservation of ethnic identity and cultures. So, the need of the hour is that the national and international authorities recognize the contribution of rural communities to the diversification of human nutrition and work in collaboration for the reappraisal of folk knowledge on WEPs.
